# Is Dosing of Therapeutic Immunoglobulins Optimal? A Review of a Three-Decade Long Debate in Europe

**DOI:** 10.3389/fimmu.2014.00629

**Published:** 2014-12-12

**Authors:** Jacqueline Kerr, Isabella Quinti, Martha Eibl, Helen Chapel, Peter J. Späth, W. A. Carrock Sewell, Abdulgabar Salama, Ivo N. van Schaik, Taco W. Kuijpers, Hans-Hartmut Peter

**Affiliations:** ^1^Section Poly- and Monoclonal Antibodies, Paul Ehrlich Institut, Langen, Germany; ^2^Department of Molecular Medicine, Sapienza University of Rome, Rome, Italy; ^3^Immunologische Tagesklinik, Vienna, Austria; ^4^Nuffield Department of Medicine, University of Oxford, Oxford, UK; ^5^Institute of Pharmacology, University of Bern, Bern, Switzerland; ^6^Hull York Medical School, University of Lincoln, Lincoln, UK; ^7^Zentrum für Transfusionsmedizin u. Zelltherapie, Charité, Berlin, Germany; ^8^Department of Neurology, Academic Medical Center (AMC), University of Amsterdam, Amsterdam, Netherlands; ^9^Department of Pediatric Hematology, Immunology and Infectious disease, Academic Medical Center (AMC), University of Amsterdam, Amsterdam, Netherlands; ^10^Centrum für chronische Immunodeficienz (CCI), University Medical Centre, University of Freiburg, Freiburg im Breisgau, Germany

**Keywords:** IVIG, SCIG, replacement therapy, immunomodulation, dosing

## Abstract

The consumption of immunoglobulins (Ig) is increasing due to better recognition of antibody deficiencies, an aging population, and new indications. This review aims to examine the various dosing regimens and research developments in the established and in some of the relevant off-label indications in Europe. The background to the current regulatory settings in Europe is provided as a backdrop for the latest developments in primary and secondary immunodeficiencies and in immunomodulatory indications. In these heterogeneous areas, clinical trials encompassing different routes of administration, varying intervals, and infusion rates are paving the way toward more individualized therapy regimens. In primary antibody deficiencies, adjustments in dosing and intervals will depend on the clinical presentation, effective IgG trough levels and IgG metabolism. Ideally, individual pharmacokinetic profiles in conjunction with the clinical phenotype could lead to highly tailored treatment. In practice, incremental dosage increases are necessary to titrate the optimal dose for more severely ill patients. Higher intravenous doses in these patients also have beneficial immunomodulatory effects beyond mere IgG replacement. Better understanding of the pharmacokinetics of Ig therapy is leading to a move away from simplistic “per kg” dosing. Defective antibody production is common in many secondary immunodeficiencies irrespective of whether the causative factor was lymphoid malignancies (established indications), certain autoimmune disorders, immunosuppressive agents, or biologics. This antibody failure, as shown by test immunization, may be amenable to treatment with replacement Ig therapy. In certain immunomodulatory settings [e.g., idiopathic thrombocytopenic purpura (ITP)], selection of patients for Ig therapy may be enhanced by relevant biomarkers in order to exclude non-responders and thus obtain higher response rates. In this review, the developments in dosing of therapeutic immunoglobulins have been limited to high and some medium priority indications such as ITP, Kawasaki’ disease, Guillain–Barré syndrome, chronic inflammatory demyelinating polyradiculoneuropathy, myasthenia gravis, multifocal motor neuropathy, fetal alloimmune thrombocytopenia, fetal hemolytic anemia, and dermatological diseases.

## Introduction

Indications for intravenous (IVIG) and subcutaneous (SCIG) immunoglobulin (Ig) therapies are steadily increasing and the annual demand has tripled in the last 15 years reaching a worldwide consumption of ~130 metric tons in 2012 (1). Shortages in supply have occurred in the past and cost pressure on health systems worldwide is growing The increased demand for therapeutic Ig has been met by increasing the number of plasma donors and by introducing new high-yield fractionation procedures, which in some cases have been accompanied by increases in rates of hemolytic anemia.

Reports involving alternative doses given in both Ig replacement and immunomodulatory indications are questioning current practice. These, together with emerging biomarkers associated with Ig-responder and non-responder status, invite close scrutiny of indications for, and methods of administering, IVIG and SCIG use in the future. Traditional dosing of Ig has relied heavily on a “per kg of bodyweight” calculation, which has now been brought into question [Ref. (2) and Chapel, submitted]. Given that IVIG/SCIG is manufactured from a limited resource, the possibility of dose adjustment according to lean body weight or even fixed doses titrated to effect in both replacement therapy and in immunomodulation arises (2, 3). This may become especially relevant in view of the combination of increasing numbers of patients due to improving diagnostics in developing countries, an aging population, and worldwide increases in body weight.

In this situation, the authors felt a need to reconsider the dosing issue for the so-called established indications (Table 1) by reviewing the literature, addressing safety and efficacy issues of new IVIG and SCIG preparations, and proposing appropriate measures where needed. The issue of class effect of different Ig preparations will be discussed. To date, switching brands during shortages or because of tender systems does not seem to have had clinically relevant effects on efficacy. However, the spectrum of side effects may differ from brand to brand, according to route of application, infusion rates, different dose levels, and the underlying disorder. In addition, product switching complicates exposure tracking in the event of a contamination incident, and alters the donor exposure profile of individual patients. Thus, dosing recommendations might have to be adjusted for certain diseases on the basis of effectiveness, safety profiles, and possibly in future on the basis of validated individual biomarkers and clinical outcome.

**Table 1:** **Well established indications according to “Guideline to assess efficacy and safety of normal intravenous immunoglobulin products” (CPMP/388/95 and CPMP/BPWG/859/95)**.

1994 “well established” indications	Dose	Frequency of injections
**REPLACEMENT THERAPY IN**		
**Primary immunodeficiency syndromes (PID)**		
• Congenital agammaglobulinemia and hypogammaglobulinemia • Common variable immunodeficiency disorders • Severe combined immunodeficiencies • Wiskott–Aldrich syndrome	Starting dose: 0.4– 0.8 g/kg – thereafter: 0.2–0.8 g/kg 0.2–0.4 g/kg	Every 3–4 weeks to obtain IgG trough level of at least 5–6 g/l Every 3–4 weeks to obtain IgG trough level of at least 5–6 g/l
**Secondary immunodeficiency syndromes (SID)**		
•Myeloma •Chronic lymphocytic leukemia (CLL) with severe secondary hypogammaglobulinemia and recurrent infections •Congenital AIDS with recurrent infections	0.2–0.4 g/kg 0.2–0.4 g/kg	Every 3–4 weeks Every 3–4 weeks to obtain IgG trough level of at least 5 g/l
**IMMUNOMODULATORY EFFECT IN**		
• Idiopathic thrombocytopenic purpura (ITP) in adults and children at high risk of bleeding or prior to surgery to correct platelet count	0.8–1 g/kg or 0.4 g/kg/day	On day 1, possibly repeated once within 3 days
• Kawasaki disease	0.4 g/kg/day	For 2–5 days
• Bone marrow transplantation (BMT)	1.6–2 or 2 g/kg	For 5 days in divided doses over 2–5 days in association with acetylsalicylic acid in one dose in association with acetylsalicylic acid

Switching from IVIG to SCIG in the case of chronic disorders has been the topic of recent research given similar efficacy to IVIG infusions, the lower incidence of systemic side effects, a lack of “wear-off” effect, improved health-related quality of life, better treatment satisfaction, and faster functional recovery with less time off work are frequently quoted advantages of SCIG (4, 5). The possible pharmacoeconomic benefits of SCIG are beyond the scope of this review, especially considering the various health/insurance systems and the varying prices of SCIG and IVIG in the different European countries. In general, switching from IVIG to SCIG in Europe is done in a dose-equivalent manner and not performed with a dose adjustment coefficient (DAC) as applied in the USA (~150%) (6, 7). As no official guidelines require a DAC in Europe (coreSPC, see below) additional product costs do not incur. The efficacy of DAC vs. dose-equivalent switch is a matter of ongoing debate and may require more long-term data.

As all authors of this review build on a long-standing professional experience in Europe, the current dosing recommendations reflect European practice, which has grown historically and encompasses recommendations from learned societies, national recommendations, European recommendations (8), and the European Medicines Agency (EMA). In the next section, the regulatory framework and the historical development of the EU recommendations will be briefly outlined without elaborating on subtle differences to American, Canadian, and Australian guidelines.

## Regulatory Framework

Three main bodies regulate blood products in Europe:

- the European Directorate for the Quality of Medicines and Healthcare (EDQM), which provides standardization of quality control of medicines as elaborated in the European Pharmacopeia; it also co-ordinates the network of the Official Medicines Control Laboratories (OMCL),
- the EMA, which evaluates marketing authorization or variation applications for medicinal products within the EU in the so-called centralized procedure (CP); it composes Investigational Guidelines for clinical trials and core Summary of Product Characteristics (coreSPCs), i.e., templates for Product Information leaflets), and
- the Heads of Medicines Agencies (HMA), which is network of the heads of the National Competent Authorities (NCA) whose organizations are responsible for the regulation of medicinal products for human use in the European economic area (EEA) in so-called mutual recognition (MR) or decentralized (DC) Procedures.

In 1994, the criteria for investigating IVIG in clinical trials were laid out in the “Guideline to assess efficacy and safety of normal intravenous immunoglobulin products” (CPMP/388/95) and a core Summary of Product Characteristics (coreSPC; CPMP/BPWG/859/95) encompassing “well established” indications for IVIG was proposed (see Table 1). These “well established” indications were based on certain pivotal studies and the doses administered therein; they became the cornerstone for the dosing recommendations in the coreSPC.

In 2000, analogous documents (investigational Guideline and coreSPC) were devised for subcutaneous and intramuscular immunoglobulin products (CPMP/BPWG/283/00 and CPMP/BPWG/282/00).

Over the years, these documents have undergone a number of revisions in order to encompass the developments in medical research and practice.

The current European Investigational Guideline and coreSPC for IVIG are undergoing a revision process (for Concept Paper see: http://www.ema.europa.eu/docs/en_GB/document_library/Scientific_guideline/2014/08/WC500170555.pdf)

The revision of Investigational Guideline and the coreSPC for SCIG is near completion: http://www.ema.europa.eu/docs/en_GB/document_library/Scientific_guideline/2012/12/WC500135705.pdf and http://www.ema.europa.eu/docs/en_GB/document_library/Scientific_guideline/2012/07/WC500130466.pdf

In the EU three IVIG and two SCIG preparations have been centrally authorized (CP procedure)^1^ and five IVIG and two SCIG brands were granted authorization via the MR- or DC-procedures^2^. In addition, some European countries will have nationally authorized products.

Several National Guidelines consider neuroimmunological diseases such as Guillain–Barré syndrome (GBS), chronic inflammatory demyelinating polyneuropathy (CIDP), multifocal motor neuropathy (MMN), myasthenia gravis (MG), and others as high to medium priority immunomodulatory indications for IVIG/SCIG use [Ref. (8); supporting information Table 2]. Progress in this field will be discussed as well as changing high-priority indications in pediatric AIDS, feto-neonatal alloimmune thrombocytopenia (FNAIT), and dermatological indications.

**Table 2:** **Abbreviations used in the text**.

BMT	Bone marrow transplantation
BPWP	Blood products working party
BWP	Biologics working party
CHMP	Committee for medicinal products for human use
CIDP	Chronic inflammatory demyelinating polyradiculoneuropathy
CLL	Chronic lymphocytic leukemia
CMD-human	Co-ordination group for MR- and DC-procedures
CMS	Concerned member states
CMV	Cytomegalovirus
CoE	Council of Europe
coreSPCs	Core summaries of product characteristics
CP	Centralized procedure
DCP	Decentralized procedure
EDQM	European Directorate for the Quality of Medicines and Healthcare
EEA	European economic area
EMA	European medicines agency
EU	European union
FNAIT	Fetal neonatal alloimmune thrombocytopenia
GBS	Guillain–Barré syndrome
GvHD	Graft vs. host disease
HMA	Heads of medicines agencies
HSCT	Hematopoietic stem cell transplantation
ITP	Idiopathic thrombocytopenic purpura
IVIG	Intravenous immunoglobulin
KD	Kawasaki’s disease
MM	Multiple myeloma
MMN	Multifocal motor neuropathy
MRP	Mutual recognition procedure
NCA	National competent authorities
OMCL	Official medicines control laboratories
PE	Plasma exchange
PAD	Primary antibody deficiency
PID	Primary immunodeficiency syndromes
PK	Pharmacokinetic
PRAC	Pharmacovigilance risk assessment committee
RMS	Reference member state
RBC	Red blood cell
SBI	Severe bacterial Infections
SCIG	Subcutaneous immunoglobulin
SID	Secondary immunodeficiency syndromes

Owing to space limitations the majority of the medium and low-priority indications [Ref. (8); supporting information Table 2] were not included in this review.

## Replacement Therapy

### Primary immunodeficiency syndromes (PID)

In 1952, Colonel Ogden Bruton (9) noted the absence of serum Ig in an 8-year-old boy with a history of pneumonia and other bacterial sino-pulmonary infections. Bruton was also the first physician to provide specific immunotherapy for this X-linked disorder by initially administering 3.2 g of IgG subcutaneously. Assuming an approximate weight of an 8-year old to be 30 kg, this dose would correspond to 0.1 g/kg. This dosing was taken into the coreSPC, but is currently considered within the low range of the recommended weekly dosing for SCIGs (0.1 g/kg–0.2 g/kg/week) or the monthly dosing of IVIG (0.4 g/kg–0.8 g/kg/month).

**Table:** 

**Current IVIG coreSPC indication and dosing:**
Primary immunodeficiency syndromes with impaired antibody production
The recommended starting dose is 0.4–0.8 g/kg given once, followed by at least 0.2 g/kg given every 3–4 weeks.
The dose required to achieve a trough level of 5–6 g/l is of the order of 0.2–0.8 g/kg/month. The dosage interval when steady state has been reached varies from 3-4 weeks. Trough levels should be measured and assessed in conjunction with the incidence of infection. To reduce the rate of infections, it may be necessary to increase the dosage and aim for higher trough levels.
**Current draft SCIG coreSPC indication and dosing:**
Primary immunodeficiency syndromes with impaired antibody production
The dose regimen should achieve a trough level of IgG (measured before the next infusion) of at least 5-6 g/l and aim to be within the reference interval of serum IgG for age. A loading dose of at least 0.2-0.5 g/kg (10-40 ml/kg) body weight may be required. This may need to be divided over several days, with a maximal daily dose of 0.1-0.15 g/kg)
After steady state IgG levels have been attained, maintenance doses are administered at repeated intervals (approximately once per week) to reach a cumulative monthly dose of the order of 0.4-0.8 g/kg. Each single dose may need to be injected at different anatomic sites.
Trough levels^a^ should be measured and assessed in conjunction with the incidence of infection. To reduce the rate of infection, it may be necessary to increase the dose and aim for higher trough levels.

^a^*N.b: Trough levels can be measured for a facilitated SCIG given every 3-4 weeks and for a normal SCIG given at biweekly-weekly intervals; however, in clinical practice SCIG products are sometimes given at even shorter intervals - in these cases the term "trough level" would not capture the fact that what is actually being measured is the mean level*.

#### Recent developments in Ig replacement therapy of PID

The demonstrated success of Ig prophylaxis via the intravenous route depends predominantly on maintaining an adequate protection against infections. According to international guidelines the Ig monthly dose of 300–600 mg/kg body weight should be administer intravenously every 3 or 4 weeks and subcutaneously once/twice a week (10–13). The trend over the past years has been to increase the monthly cumulative doses (14–17). This general rule might not be optimal for all patients affected by primary antibody deficiencies (PAD) due to high clinical and immunological heterogeneity of the underlying diseases. A recent paper (18) analyzed the clinical presentation, association between clinical features, and differences and effects of Ig treatment in a large series of European patients affected by common variable immunodeficiency disorders (CVID), the most common symptomatic PAD. Different treatment strategies applied in Europe resulted in considerable differences in Ig dosing, ranging from 0.13 up to 0.75 g/kg/month. This and previous studies suggested that a correlation between patients’ antibody levels and clinical effects: patients with very low-trough levels of <4 g/l had poor clinical outcomes (15, 16, 19) whereas higher trough levels were associated with a reduced frequency of serious bacterial infections.

Thus, the aim should be to maintain an individual’s effective antibody level and not to establish a universally defined immunoglobulin monthly dosage. Consequently, almost all recent studies on Ig replacement advocate that the treatment strategies should be individualized not only in terms of dosages but also with regard to treatment schedules including intervals between administrations and routes of administration (20–23). Milito et al. (24) have recently demonstrated that in PAD patients with fewer disease-associated complications the IVIG replacement could be administered with the widely used interval of 3 or 4 weeks, even administering low-IVIG replacement dosages. On the other hand, in patients with bronchiectasis and enteropathy and a severe immunological phenotype with IgG trough levels <500 mg/dl, IgA <7 mg/dl, absent response to polysaccharides, and low-switched memory B cells (<2%) the protective effect might be achieved by lowering the interval between administrations to 2 weeks and in few cases to 1 week, without increasing the cumulative monthly Ig dosage (19). Alternatively, patients with severe clinical and immunological phenotypes might be treated with higher Ig dosages at an interval of 3–4 weeks. The need to elucidate the effects of therapy on patient outcomes might allow identification of what works best in which setting and under what conditions (25, 26). Health care delivery systems are quickly changing in response to economic pressure and concerns about quality of care emerge. The system of care is itself an important determinant of patient outcomes. The promise of individualized medicine has launched a huge research enterprise to explore the personal characteristics that influence responses to therapy.

The pharmacokinetics (PK) of IgG and specific antibodies exhibited a different half-life in patients treated at different intervals between infusions (26). Trough levels of the lowest specific antibody concentrations rise if regular infusions are given and the actual trough levels in a regularly infused patient are likely to be higher than the levels of specific IgG measured by ELISA in the IVIG preparations (27, 28).

The clinical relevance of regular application of Ig has been underlined by the finding that an important determinant of the efficacy of Ig prophylaxis is the length of time an individual spends with a lower IgG level a situation, which is minimized by subcutaneous therapy (29). This time is more dependent on the patient’s IgG half-life and the frequency of dosing than on the dose of Ig infused. In clinical practice, it would be ideal to perform a PK study in all patients. However, this would require a significant commitment in time and costs from the patient and the treating physician. This practical drawback has limited the use of PK information in clinical practice. Different alternative options have been attempted. The Oxford approach, for example, based on monitoring break-through infections, was to increase the IVIG dose by 0.15 g/kg/month when patients present with a serious infection, or three or more moderate infections over a year (17). This recommendation could be an alternative for patients who have persistent infections; although other factors such as protein loosing conditions, airway and intestinal inflammation, need to be assessed when defining an individual Ig treatment schedule as the Oxford approach related treatment to CVID phenotype (17). Moreover, in PAD, several lines of experimental evidence gathered recently (30–32) provided a basis for an active role for IVIG in immunomodulation beside the main role to replace the missing antibodies. Ig has such a role in regulating autoimmune and inflammatory responses through modulating B and other cells functions (8). These new findings might help to explain the different results found in trials aimed at establishing the clinical outcome of Ig replacement in PAD patients. It is possible that some of the positive effects observed in patients treated with higher doses are not dependent only on the prophylactic role of Igs but also on their anti-inflammatory ones. In contrast to subcutaneous administration, intravenous administration might allow maintenance of the protective and immunomodulatory effects due to the serum IgG peak reached at the time of each administration.

Methods to improve IgG recovery and increase productivity have been implemented in the last few years as a response to growing clinical demand for therapeutic Igs (27). Any effects of major changes in the Ig production should be assessed in clinical and drug surveillance studies.

In addition, approval has been granted for

- high-concentration formulations of IVIG and SCIG preparations (27, 29, 33),
- rapid push administration (34),
- one SCIG product (Hyqvia) combined with the prior use of a spreading factor (hyaluronidase), thus, allowing for 3–4 weekly intervals (current indication excludes children <18 years) (35)^3^, and
- one SCIG product (Hizentra) where modeling and simulation approaches were applied that led to comparable IgG exposure levels if the product were to be administered every 2 weeks using double the weekly dose during maintenance therapy (21, 22)^4^.

#### SCIG studies in PID

A recent evidence-based review article (36), comparing IVIG and SCIG administration in PID and SID patients, encompassed 25 studies (in PID patients 2/25 studies were randomized and 17 non-randomized; for SID patients 1/25 studies was non-randomized and 5/25 were health economic studies). Of the studies that mentioned specific products, eight used an SCIG from CSL-Behring, seven a product from Baxter, and two a product from Grifols. Only 3/25 studies reported severe bacterial infections (SBI) as their primary outcome of interest for both SCIG and IVIG; no SBI occurred in these studies. In a total of five studies, the annual number of infections was investigated and no difference found between the two routes of administration; however, the definitions of infections were fairly heterogeneous. Higher IgG trough levels were found with SCIG substitution. No serious adverse events were reported in five trials that investigated this parameter, however, here again definitions and inclusion/exclusion criteria varied. Minor adverse events, consisting of local symptoms were usually mild and more frequent with SCIG substitution, as would be expected of this modality. Four studies investigating health-related quality of life showed improvement when patients switched from hospital-based IVIG to SCIG substitution at home. Of the 5/25 studies that evaluated health economics, 4 found that SCIG administration was considerably more cost effective in comparison with IVIG substitution, whereas one older study did not show this gain.

The authors caution the reader that for most efficacy and safety parameters measured in the studies the value of evidence was low and that with regard to the pharmacoeconomic evaluation due to the differences in the health care and insurance systems in the different countries the results cannot be generalized. They conclude that good studies comparing IVIG to SCIG are lacking, but it is possible to state that SCIG is safe and efficacious and at least non-inferior to IVIG. They also view the issue of switching from IVIG to SCIG as not yet solved.

### Secondary immunodeficiency syndromes (SID)

Patients with secondary immunodeficiency are a continuously increasing heterogeneous group (36, 37). Recommendations for treatment of antibody deficiency associated with lymphoid malignancies [e.g., CLL, multiple myeloma (MM)], post-hematopoietic stem cell transplantation (HSCT), or HIV are referred to in core SPCs, which require critical updates. Those SIDs due to medications [e.g., anticonvulsants, anti-rheumatics, MAbs (e.g., anti-CD20), chemotherapy, immunosuppression] may be suspected from the patient’s medical history although usually only a subset of patients develops signs of SID.

SID patients are often less well defined than PADs as the diagnosis relies only on serum Ig levels rather than proven antibody failure, but their clinical importance is recognized increasingly. In this section, findings in patient groups, including those not included in the core SPC, are discussed.

Patients with SID are unduly susceptible to different types of pathogens depending on the type of SID: viral and/or opportunistic infections are common in patients with non-functional or absent T-cells while bacterial infections predominate in antibody failure syndromes. Severe life-threatening infections affect the respiratory or gastrointestinal tracts though chronic infections of prolonged duration also occur with agents of low pathogenicity (38, 39). These infections are an important cause of morbidity, mortality, hospital admissions, and intensive care treatment in this group of patients. The following discussion relates largely to antibody failures secondary to therapy or disease that maybe amenable to treatment with Ig replacement therapy.

Numerous studies in patients with lymphoid malignancies have shown beneficial effects of IVIG, resulting in fewer infectious episodes, reduced use of antibiotics, shorter hospital stay but no difference in overall mortality. Most of these studies report on patients in chronic state of disease, treated with IVIG doses comparable to patients with PAD.

#### Chronic lymphocytic leukemia (CLL)

In 1988, the Cooperative Group for the Study of Immunoglobulin in CLL published the results of a double-blind, placebo-controlled study (40) in 81 CLL patients (with 50% lower than normal IgG levels and a history of >1 serious infection since onset of the illness). Those patients who received immunoglobulin therapy (0.4 g/kg every 3 weeks for ~1 year) had significantly fewer bacterial infections compared to those in the placebo group.

**Table:** 

**Current IVIG coreSPC indication and dosing:**
Hypogammaglobulinaemia and recurrent bacterial infections in patients with CLL, in whom prophylactic antibiotics have failed.
The recommended dose is 0.2–0.4 g/kg every 3–4 weeks.
Current draft SCIG coreSPC indication and dosing:
Hypogammaglobulinaemia and recurrent bacterial infections in patients with CLL, in whom prophylactic antibiotics have failed or are contra-indicated.
For dosing see PID/SCIG above.

Following the original multi-center, randomized, placebo-controlled study (40) that resulted in inclusion in the coreSPC, the protective effect of IVIG in CLL and low-grade non-Hodgkin’s lymphoma was confirmed by a 2 year, placebo vs. Ig cross-over study (41); in this study, serious bacterial infections correlated with low-serum IgG levels (<6.4 g/l) (41). Along the same line Boughton et al. (42) noted that 10 patients (24%) with IgG levels <3.0 g/l experienced 65% of infections in the whole CLL group. In view of newer treatments for CLL, studies on frequencies of bacterial infections before and after treatment with fludarabine, anti-CD20, or BTK inhibitors will be important. Such studies need to include documentation of IgG levels and specific antibody titers following test immunization.

Antibody deficiency, for which hypogammaglobulinemia is a surrogate, is the most important risk factor for bacterial infections; furthermore, antibody failure, as shown by test immunization, can occur in the absence of hypogammaglobulinemia (43). Failure to respond to test immunization with approved, killed vaccines is the most reliable indicator though absence of circulating antibodies after documented infections, such as HSV or VZV, are also helpful. Test immunizations in CLL patients have shown poor IgG responses against a range of antigens, including polysaccharide vaccines or conjugate pneumococcal vaccines (44). Whether those patients that do produce antibodies are even protected against bacterial infections is not clear due to lack of clinical trials (45). At present most guidelines use serum IgG levels in SID but logic requires test immunizations of patients (one protein and one polysaccharide vaccine) rather than relying on previous infections or surrogate markers.

Data from the original trials (40, 41) showed that starting doses of IVIG used in PAD (0.4 g/kg/month) were protective against bacterial infections in patients with low-serum IgG levels. Protection at a lower dose of IVIG (0.3 g/kg/month) was confirmed by Molica et al. (46) in a cross-over study in which 42 patients with serum IgG levels <6 g/l and/or a history of severe infection received either IVIG (0.3 g/kg/month) or standard care for 12 months and then crossed over to the alternative regime. Jurlander et al. (47) showed that 15 patients with low-serum IgG receiving 10 g IVIG every 3 weeks (0.2 g/kg/month) had reduced hospital admissions for infections and significantly reduced febrile episodes although neither were totally abolished.

#### Multiple myeloma (MM)

In 1994, Chapel et al. published a study on IVIG as a prophylaxis in 83 patients with stable MM (48). As in the case of CLL, the administration of IVIG (0.4 g/kg every 4 weeks for 1 year) led to significant reduction in life-threatening infections compared to placebo.

**Table:** 

**Current IVIG coreSPC indication and dosing:**
Hypogammaglobulinaemia and recurrent bacterial infections in plateau phase MM patients who have failed to respond to pneumococcal immunization.
The recommended dose is 0.2–0.4 g/kg every 3–4 weeks.
Current draft SCIG coreSPC indication and dosing:
Hypogammaglobulinaemia and recurrent bacterial infections in MM patients
For dosing see PID/SCIG above.

The randomized placebo-controlled multi-center study in plateau-phase MM patients given IVIG 0.4 g/kg monthly for 1 year showed significant reductions in frequency and severity of infections (48). Fortunately, this trial included test immunization with polysaccharide pneumococcal vaccine before starting Ig therapy and benefit from IVIG was shown only in patients who responded poorly to pneumococcal immunization and had normal numbers of circulating neutrophils (48). Recently, antibody failure to a range of pathogens in MM and related conditions has been confirmed (49) suggesting that IVIG therapy may be justified. However, many newer treatment regimes in MM use antibiotic prophylaxis and each patient should be considered primarily on an individual basis for Ig therapy until more data is provided. Similar conclusions were drawn from a recent systemic review and meta-analysis by Raanani et al. (50) suggesting that IVIG cannot be recommended routinely for patients with CLL or MM with hypogammaglobulinemia and/or recurrent infections; instead decisions should be made on an individual basis.

#### Bone marrow transplantation

In 1990, Sullivan et al. (51) published the results of a double blind, placebo-controlled study in 382 bone marrow transplant recipients. The patients received placebo or 0.5 g/kg IVIG from day −7 to day +90, and continued IVIG treatment on a monthly basis until 1 year after transplantation. The endpoints were the rate of interstitial pneumonia and acute Graft vs. Host Disease (GvHD). Among the 61 cytomegalovirus (CMV) seronegative patients, none contracted interstitial pneumonia and in the group of 308 CMV positive patients the IVIG treated ones fared significantly better. Acute GvHD was reduced in the IVIG treated patients over 20 years of age but not in the younger age group.

In the initial coreSPC, the indication covered both GvHD and infection prophylaxis (mainly for CMV infection) and was thus placed in the category of immunomodulation. However, a later placebo-controlled, double-blind study by Cordonnier et al. (52) and a meta-analysis on 30 trials including 4223 patients (53) revealed that there was no benefit on survival and frequency of infections while the risk of veno-occlusive disease (VOD) was increased. Thus, after allogeneic HSCT IVIG is currently used as a replacement therapy for hypogammaglobulinaemic patients with secondary B cell deficiency (serum IgG < 4 g/l) and no longer for GvHD.

The use of SCIG compared to IVIG has been studied in one non-randomized, retrospective study in 58 children with prolonged hypogammaglobulinemia after HSCT. All children were treated with IVIG and 12 continued on SCIG home treatment (54). While being of equivalent efficacy to IVIG, SCIG showed fewer side-effects and was the preferred treatment option.

**Table:** 

**Current IVIG coreSPC indication and dosing:**
Hypogammaglobulinaemia in patients after allogeneic haematopoietic stem cell transplantation (HSCT).
The recommended dose is 0.2–0.4 g/kg every 3–4 weeks. Hypogammaglobulinaemia in patients requiring allogeneic hematopoietic stem cell transplantation (HSCT).
For dosing see PID/SCIG above.

#### Congenital AIDS

In the late 1980s, the US National Institute of Child Health and Human Development (NICHHD) performed a double blind, placebo-controlled, multi-center IVIG study in 372 children with symptomatic HIV infection (55). The children were given 0.4 g/kg every 4 weeks. Median follow-up time was 17 months. The results showed that for children with CD4+ T-cell entry values >200/ml the time free of serious bacterial infection increased, serious and minor infections were reduced, as were the number of hospitalizations. However, there was no survival advantage for the IVIG group.

A further double blind, placebo-controlled IVIG study in 255 children with AIDS was published in 1994 (56). All children had received 180 mg/m^2^ of zidovudine. The IVIG dose was 0.4 g/kg every 4 weeks. Patients were stratified according to their history (>1 serious bacterial infection), previous zidovudine treatment, and trimethoprim–sulfamethoxazole (TMP–SMZ) prophylaxis for *Pneumocystis jirovecii* pneumonia at entry. Median follow-up time was 30.6 months. The children who were on IVIG plus zidovudine and not receiving TMP–SMZ prophylaxis had a reduced rate of serious bacterial infections. As currently an efficient, highly active anti-retroviral therapy (HAART) is given to the mothers prior to delivery the development of congenital AIDS and pediatric HIV associated antibody deficiency is greatly reduced. In these patients, IVIG treatment is medically necessary for the prevention of bacterial infection when the following criteria are met (A) diagnosis of HIV disease, (B) patient age ≤13 years, and (C1) documented hypogammaglobulinemia or (C2) functional antibody deficiency as demonstrated by pool specific anti-titers (or recurrent bacterial infections). IVIG dose should not exceed 1.4 g/kg every 28 days. IVIG replacement therapy in HIV infected children without antibody deficiency may not be necessary; indeed it is even contra-indicated in the UK Guidelines.

**Table:** 

**Current IVIG coreSPC indication and dosing:**
Congenital AIDS with antibody deficiency and recurrent bacterial infections.
The recommended dose is 0.2-0.4 g/kg every three to four weeks.
No current SCIG coreSPC indication or dosing under discussion.

#### Recent developments in Ig replacement therapy of SID

##### After anti-CD20 therapy

Anti-CD20 trials, however, have monitored both circulating B cells and serum Igs and although originally reduction of serum Igs was thought to be transient, some patients continue to have hypogammglobulinaemia and accompanying infections for prolonged periods possibly forever (57). In this recent study from Sloan-Kettering involving patients with lymphoid malignancies, low-serum IgG levels were identified in 38.5% (69/179) of patients after CD20 therapy, all of whom had normal levels initially; the risk was greater in patients who received maintenance rituximab. In 14 patients of this subset, IVIG significantly reduced the frequency of sino-pulmonary bacterial infection and pneumonias (57). Likewise, monitoring serum IgG levels and B cell numbers after anti-CD20 treatment in ANCA+ vasculitis is warranted for recognition of SID (58, 59), which may require IVIG replacement therapy.

##### After immunosuppressive regimes in solid organ transplantation

Floruesco et al. (60) discuss the impact of hypogammaglobulinemia on the rate of infections and survival following solid organ transplantation, in a meta-analysis that included 1756 patients from 18 studies. The study included patients with lung, kidney, heart, and liver transplants. The rate of severe hypogammaglobulinemia (IgG < 0.4 g/dl) amounted to 15%; it significantly increased the risk of CMV, fungal, and respiratory infections and was associated with higher 1-year all-cause mortality as originally described by Rubin (61). Sarmiento et al. (62) looked at 75 patients post heart transplantation of whom 10 patients developed CMV disease; those with a low-serum IgG level (IgG < 5 g/dl) were at higher risk of reactivation of CMV disease; the authors recommended CMV monitoring as a potential tool to recognize high-risk patients (62). More recently, Carbone et al. (63) reported results from 55 consecutive adult heart recipients who were subjected to an immune monitoring including measurement of specific antibodies and underwent IVIG therapy when SID was established. Eighty five percent of severe infections occurred during the first 3 months and mean time to IVIG infusion was 2.47 months. IVIG therapy resulted in improved specific antibody titers in the group on replacement therapy and a significant reduction of bacterial infections, in the substituted group of patients compared with 55 untreated patients.

##### During autoimmune diseases

An increased risk of invasive pneumococcal disease has been demonstrated in a retrospective analysis of a cohort of systemic autoimmune diseases including patients with SLE, rheumatoid arthritis, hemolytic anemia, and Sjogren’s syndrome (37–39, 64). In a 10-year European study of 1000 SLE patients 68 patients died (6.8%) and the most frequent causes of death were similarly divided among active SLE (26.5%), thrombosis (26.5%), and infections (25%), especially in the first 5 years after diagnosis (65). Similar results have been obtained in a multi-ethnic US cohort study: the 5-year mortality was 11.8%, active SLE (41%), and fatal infections (32%) headed the ranking (66). Infections were also frequent causes of increased hospitalization (67). Prolonged hypogammaglobulinemia requiring antibody replacement therapy occurred in 21% of ANCA+ vasculitis patients treated with cyclophosphamide followed by rituximab (58).

##### Antibody failure due to medications other than immunosuppressants

It has been known for a long time that anticonvulsant therapy with phenytoin or carbamazepine can cause low-serum IgG levels and recurrent infections (68, 69). Recently, valproic acid, a histone deacetylase (HDAC) inhibitor, has also been demonstrated to inhibit early B cell differentiation and activation (70) leading to hypogammaglobulinemia. Few studies performed in small patient groups with SID due to immunosuppressive medication or chemotherapy also demonstrate beneficial effects of IVIG treatment. However, robust studies looking on clinical outcome are lacking, not least as prophylactic antibiotics are used as standard measure in many centers.

Most studies in SID conditions such as CLL, MM, solid organ transplantation, and autoimmune diseases have been performed in chronic, stable disease with 0.3–0.4 g/kg IVIG every 3–4 weeks. Outcome in patients with antibody deficiency, with or without low-serum IgG levels as well as episodes of severe – potentially lethal – infection have to be included in the analysis (37–39). Present research in translational medicine including PID aims at early diagnosis to identify patients before the first (potentially life threatening) infectious complications and this applies to SID as well. Further clinical trials have been recommended in patients with SID. Patient selection in such studies will be critical and only test-immunized patients with proven antibody deficiency should be entered (one T dependent vaccine – usually tetanus toxoid is used since the assay is reliable and available in immunology laboratories and one T-independent vaccine – either pneumovax or polysaccharide *Salmonella* vaccine – both with reliable assays as used in PIDs). IVIG should be given early on during the course of an aggressive immunosuppressive therapy since lethal infections often occur at this stage.

## Immunomodulatory Therapy

### Idiopathic thrombocytopenic purpura

In 1981, Paul Imbach and colleagues (71) treated 13 children with idiopathic thrombocytopenic purpura (ITP) (6 with acute and 7 with chronic forms of ITP, platelets counts <30,000/μl) with 0.4 g/kg/day IVIG for 5 days and could demonstrate a normalization of platelets counts (150–600 × 10^3^/μl) in 12/13 children within 5 days. However, the effect was transient as platelets fell to 80–400 × 10^3^/μl during the following 10 days.

In the years after this study, various trials followed in adults with ITP – also comparing IVIG to prednisone. In a study by Blanchette et al. (72), a reduced dose of 0.8–1.0 g/kg was given on day 1. If 48–72 h later platelet counts remained at values ≤20 × 10^3^/μl a second IVIG dose was recommended. Although this protocol proved to be equally efficacious as the original of Imbach et al. (70), it was the latter one that set the stage for the regulatory adoption of the dosage regimen in ITP and other “immunomodulatory settings” for years to come.

In 2009, the International Working Group (73) standardized the terminology, definitions, and outcome criteria for clinical trials in ITP, which in turn was taken on board during the IVIG guideline and coreSPC revision process.

**Table:** 

**Current IVIG coreSPC indication and dosing:**
Primary immune thrombocytopenia (ITP), in patients at high risk of bleeding or prior to surgery to correct the platelet count.
There are two alternative treatment schedules:
0.8–1 g/kg given on day one; this dose may be repeated once within 3 days
0.4 g/kg given daily for 2–5 days.
The treatment can be repeated if relapse occurs.
No current SCIG coreSPC indication or dosing under discussion.

#### Recent developments in ITP research

Treatment options in ITP vary with patient age (childhood vs. adults) and diagnostic status (newly diagnosed, persistent for 3–12 months, chronic beyond 12 months, or secondary to other diseases) (74, 75). Notably, in newly diagnosed ITP of childhood (<18 years) the standard of care is still IVIG at a total dose of 0.8–1.0/kg given on 1 or 2 consecutive days. In IVIG non-responders, the results can be improved by adding 20 mg/kg methylprednisolone during day 1–3 (75). In a prospective randomized trial, a 2 g/kg total dose IVIG was clearly more effective than 50 or 75 μg/kg anti-D as first-line treatment in childhood ITP (76). Interestingly, individual cases of successful treatment with very low-IVIG doses (100 or 200 mg/kg) imposed by economic constraints have been reported (77, 78). These observations warrant a systematic evaluation of an up-scaling protocol starting with doses of 0.2–0.4 g/kg IVIG. In a study from Thailand (a developing country), cost effectiveness of IVIG in childhood ITP has been proven, as compared to standard treatment of thrombocyte transfusions, corticosteroids plus immunosuppressants (79). On the other hand, health economic studies from Canada and Ireland show for adult chronic ITP patients that romiplostim, a thrombopoietin receptor agonist, seems to compare favorably with standard treatment including IVIG (80, 81).

A general observation throughout all ITP studies is an IVIG response rate of 60–75% in newly diagnosed childhood ITP. Hopefully, new biomarkers may in future be able to identify early on IVIG responders from non-responders. Thus, a recent study by Morimoto et al. (82) indicates that patients with WBC count <7.0 × 10^9^/l had a lower probability of thrombocytopenia-free survival (41 vs. 77%, *P* = 0.003) and a higher rate of progression to chronic ITP (29 vs. 6%, *P* = 0.040) than those with WBC count ≥7.0 × 10^9^/l. These results suggest that ITP with lower WBC count may represent a distinct subgroup requiring early on additional or other treatments than IVIG [e.g., rituximab (83)]. Similarly, in adults with ITP, the presence of anti-GPIb-IX auto-antibodies is a predictor for poor response to IVIG treatment: only 36.4% responded as compared to 80% of anti-GPIb-IX negative ITP patients (84). Less promising results came from studies correlating FcγRIIa and FcγRIIIa polymorphic variants to IVIG responsiveness and outcome (85): while the high-affinity FcγRIIIa variant 158V is possibly implicated in the pathogenesis of ITP, FcγRIIa (131R), and FcγRIIIA (158V) variants do not seem to impact on chronicity and therapeutic efficacy of IVIG, although studies on such correlations are underway. In this respect, it is interesting that the expression of an open-reading frame for the activating FcγRIIc (instead of the more common, non-expressed pseudogene) also seems to predispose to ITP (86).

### Kawasaki disease

Kawasaki disease (KD) is an acute self-limiting inflammatory disorder of children, associated with vasculitis, affecting predominantly medium-sized arteries, particularly the coronary arteries. In 1984, Furusho et al. (87) treated 93 patients with KD [45 with acetylsalicylic acid (ASA) and 40 with ASA + IVIG] at a dose of 0.4 g/kg/day for 5 day. They observed a greater percentage of patients with coronary artery lesions in the ASA-alone group compared to the combined therapy (42 vs. 15%).

In 1991, Newburger et al. (88) treated 276 KD patients with ASA and IVIG 0.4 g/kg/day for 4 day vs. 273 ASA + IVIG 2 g/kg (given once). The rational to choose 2 g/kg (and not 1.6 g/kg) for the single dose was that serum IgG concentration on day 4 of the 0.4 g/kg × 4 day regime would be approximately the same. It was shown that the single large dose was more effective than the conventional regimen and equally safe. Both dosing possibilities were taken into the coreSPC (0.4 g/kg/day IVIG for 5 day + ASA and IVIG 2 g/kg + ASA).

**Table:** 

**Current IVIG coreSPC indication and dosing:**
Kawasaki disease.
2.0 g/kg as a single dose is the recommended treatment worldwide. Patients should receive concomitant treatment with acetylsalicylic acid.
No current SCIG coreSPC indication or dosing under discussion.

#### Recent developments in KD research

The etiology of KD is still unknown. It is assumed that unidentified infectious agents trigger a strong, self-limiting inflammation in genetically susceptible hosts. Numerous studies have been undertaken to identify susceptibility genes for KD as well as for resistance to IVIG treatment. Polymorphic variants of *FCGR2A, CD40, ITPKC, FAM167A-BLK*, and *CASP3* have been shown to be associated with KD (89). Similarly, gene copy number (GCN) variants of FcγR2c and FcγR3b were significantly associated with KD susceptibility and seem to influence also the IVIG treatment response (90) as does the increased expression of IL-1 pathway genes (91). Recently, Ogata et al. (92) found that sialylation levels of therapeutic IVIG are unrelated to treatment response whereas low sialylation of endogenous IgG and low-serum β-galactoside:α2-6 sialyltransferase-I (ST6Gal-I), ST6GAL1 RNA, and enzyme levels predict therapy resistance. As the authors compare only 10 IVIG responders to 10 non-responders their findings have to be met with great caution and need a rigorous confirmation.

The analysis of 3860 data sets from children with KD registered in the Taiwan National Health Insurance Data Base focused on the impact of different IVIG manufacturing procedures on the responsiveness in KD (93). They compared effects of β-propiolactone, acidification, and IgA content. Whereas β-propiolactone treatment of Ig had a relative risk of 1.45 to confer IVIG non-responsiveness and prolonged anti-platelet and anti-coagulants treatment, the relative risks for acidification and IgA content were non-significant in this respect. These findings are difficult to confirm as IVIG treated with β-propiolactone is no longer on the market.

On clinical grounds IVIG non-responders were shown to be older, had >6 days fever before the initiation of IVIG therapy; their serum levels of CRP, total bilirubin, lactate dehydrogenase (LDH), and gamma-glutamyltranspeptidase (g-GT) were significantly higher (*P* = 0.002, *P* < 0.001, *P* < 0.034, and *P* < 0.038, respectively), and their hemoglobin value was significantly lower (*P* = 0.025) than in IVIG responders (94–96). The authors defined the following predictors for IVIG non-responders: CRP level >10 mg/l, LDH level >590 IU/l, and/or hemoglobin value <10 g/l and suggested as escalating treatment options corticosteroids (97), TNF blockers (89, 98, 99), or plasma exchange (100).

Until relatively recently, corticosteroids were considered potentially detrimental in KD, as early studies showed an association with worse outcome (101). However, it is likely that this at least in part reflected an inadvertent selection bias, as those with more severe KD received corticosteroids. Corticosteroids are recommended as “rescue” therapy if there is no response to initial infusion(s) of IVIG (102). More recently, the potential role of corticosteroids as adjunct primary therapy in addition to IVIG has been addressed in randomized trials, either in unselected patients or in those considered at particularly high risk of coronary artery damage.

A multicenter, randomized, double-blind trial from the U.S. assessed primary treatment with IVIG (2 g/kg) and aspirin with or without a single dose methylprednisolone (30 mg/kg) in 199 unselected children. Addition of a single steroid dose to conventional therapy did not improve coronary artery outcomes (103).

A more recent prospective randomized, open-label, trial in Japan enrolled only those assessed by a locally derived risk score as being at particularly high risk of coronary damage (104). Patients were randomized to a prolonged course of intravenous followed by oral prednisolone (or placebo), in addition to standard therapy with IVIG (2 g/kg) and aspirin. Coronary artery outcomes during the 4-week study period were significantly better in the corticosteroid group. However, the generalizability of these findings is uncertain; in particular, the scoring system on which selective recruitment was based does not perform well in non-Japanese patients (105, 106). As approximately three-quarters of KD patients were excluded, as they did not meet enrollment criteria [including those with coronary artery dilatation at presentation (104)], it remains unclear whether this corticosteroid regimen would benefit KD patients more broadly. Moreover, the prolonged intravenous course of corticosteroids would itself incur significant additional costs by prolonged admission and potential side effects (105).

### Guillain–Barré syndrome

In the late 1990s, the indication GBS was taken on board the coreSPC as a new “established indication” after a prior authorization of a product specific indication within a variation procedure. The variation procedure showed that the data were mainly based on three published studies which each used different Ig products but revealed similarly efficacious outcomes with regard to decrease in disability grading when compared to plasma-exchange (PE) – the standard therapy at the time (107–109). In the studies by van der Meché (107) and the GBS Trial Group (109), the dosing was 0.4 g/kg × 5 day and in the study by Bril et al. (108) 0.5 g/kg × 4 day. The dosing taken into the coreSPC was 0.4 g/kg × 5 day.

**Table:** 

**Current IVIG coreSPC indication and dosing:**
Guillain–Barré syndrome.
0.4 g/kg/day over 5 days.
No current SCIG coreSPC indication or dosing under discussion.

#### Recent developments in GBS research

Guillain–Barré syndrome is characterized by several subtypes (110). The most common form, acute inflammatory demyelinating polyneuropathy (AIDP), is characterized by segmental demyelination in peripheral nerves with acute flaccid paralysis. An axonal variant without demyelination either in the form of acute motor axonal neuropathy (AMAN) or acute motor and sensory axonal neuropathy (AMSAN) have been distinguished from AIDP. The Miller Fisher syndrome (MFS) variant is defined by the clinical triad of ophthalmoplegia, areflexia, and ataxia. High titers of anti-ganglioside IgG auto-antibodies have been described in GBS: anti-QD1a/Anti-GM1 IgG in AMAN, and Anti-QD1b IgG in MFS. Antecedent infections (*Campylobacter jejuni, Mycoplasma pneumoniae*, or EBV) support the hypothesis of a “carbohydrate mimicry” driven immunopathogenesis.

N-glycosylation of the Fc-portion of serum IgG was investigated in patients with GBS before and after treatment with IVIG in relation to clinical course and outcome (111). Treatment-naive GBS patients compared with age- and sex-matched controls had lower levels of galactosylation of IgG1 and IgG2. IVIG preparations contained relatively high levels of galactosylated and sialylated IgG Fc glycoforms compared with serum IgG in patients. Treatment with IVIG resulted in an increase in serum of the Fc-galactosylation and -sialylation of both IgG1 and IgG2. Multiple logistic regression analysis showed that patients with persistent low-IgG galactosylation and sialylation despite IVIG treatment had the most severe forms of GBS and needed ventilator support more often.

Guillain–Barré syndrome normally runs a monophasic disease course and immunomodulatory treatment is only needed during the acute phase of the disease. PE and IVIG are both proven to be equally effective in GBS, while corticosteroids do not confer any benefit (112–114).

The empirical dose of IVIG is 0.4 g/kg/day for 5 days, which is based on practice in other autoimmune diseases. In a small, randomized trial including 39 GBS patients, treatment with 0.4 g/kg/day for 3 or for 6 days was compared. In patients receiving six treatments, there was a non-significant trend toward a better outcome. This finding became significant in ventilated patients (115). In another randomized, open trial with 51 children with GBS 1.0 g/kg/day IVIG for 2 days was compared with 0.4 g/kg/day for 5 days, giving the same total dose to each (116). There were no significant differences in the primary or secondary outcome measures except that early relapses were significantly more common after the 2-day (5/23) than the 5-day regimen (0/23; *P* = 0.049) In one study, including 50 GBS patients, the total dose of 2 g/kg was given over 4 days and compared with plasma exchange. Both treatments were equally effective; IVIG had less adverse events (108).

Interestingly, the increase in serum IgG (ΔIgG) 2 weeks after IVIG treatment varied considerably (mean 7.8 g/l SD 5.6 g/l). Patients with low-ΔIgG recovered significantly more slowly and fewer could walk unaided at 6 months (log-rank *P* < 0.001) (117).

In the latest Cochrane Review of seven trials with a variable bias risk, IVIG was compared with PE in 623 severely affected participants. In five trials with 536 participants for whom the outcome was available, the mean difference of change in a seven-grade disability scale after 4 weeks was not significantly different between the two treatments (118). The authors concluded that IVIG when started within 2 weeks of disease onset hastens neurological recovery as much as PE. Adverse events were not significantly different with either treatment but IVIG was significantly more likely to be completed than PE. IVIG following PE did not provide significant additional benefit. GBS patients receiving a combination of IVIG and glucocorticosteroids did not recover faster than patients receiving IVIG alone (119) and adding mycophenolate mofetil to a combined treatment with IVIG and corticosteroids did not improve outcomes (120). In a small pilot study, adding IFN-β1a to IVIG treatment did not contribute to a better outcome (121).

Various studies have found that IVIG may be superior to PE, especially in GBS patients with a preceding *C. jejuni* infection and GM1 or GM1b auto-antibodies. However, none of these correlations is strong enough to guide therapeutic decisions (122). Clearly, more dose finding and biomarker research is warranted in all forms of GBS including pediatric GBS.

### Chronic inflammatory demyelinating polyradiculoneuropathy

Eight randomized controlled trials (RCT) including 332 CIDP patients using different IVIG brands and comparing the effects to either placebo, prednisone or PE showed that IVIG improves disability short-term, in one large trial the benefit of IVIG persisted for at least 24 weeks (123). Currently, five IVIG products are licensed for CIDP either purely nationally or via the MR-procedure in certain EU states or, in one case, centrally in the entire EU.

**Table:** 

**Product specific indication and dosing:**
Chronic inflammatory demyelinating polyradiculoneuropathy (CIDP)
For those IVIG products which have the indication CIDP, the dosing generally consists of a loading dose at 2 g/kg (given over 2–5 days) followed by maintenance doses of 1 g/kg (given over 1–2 days), cautioning physicians that the duration of treatment beyond 24 weeks should be subject to their discretion based upon the patient’s response and maintenance response in the long-term and further that the dosing and intervals may have to be adapted according to the individual course of the disease.
No current IVIG/SCIG coreSPC indication or dosing available.

#### Recent developments in CIDP research

Chronic inflammatory demyelinating polyneuropathy is an immune-mediated peripheral nerve disorder characterized by motor and/or sensory symptoms and signs in more than one limb, developing over at least 2 months. The disease runs a progressive, relapsing-remitting, or monophasic course and can lead to significant disability due to walking difficulties and loss of arm dexterity. A diagnosis relies heavily on electrophysiological studies that typically show evidence of conduction block and demyelination. Apart from the typical clinical picture, the EFNS/PNS CIDP treatment guideline has defined several atypical CIDP phenotypes of which the pure sensory form is most frequently occurring. The atypical forms of CIDP may exhibit a different natural course and treatment response (124).

The key mechanisms in the pathogenesis have not been identified although several studies have highlighted the role of T-cells in CIDP and an important role for auto-reactive T-cell responses against peripheral myelin antigens such as P0, P1, P2, and peripheral myelin protein (PMP)-22 has been suggested (125–130).

The short- and midterm efficacy of corticosteroids, IVIG, and PE has been demonstrated in CIDP in several RCTs and meta-analyses (131–138). The most recent Cochrane systemic review (123) analyzed 8 RCTs with a total of 332 eligible patients. The authors concluded that IVIG improves disability for at least 2–6 weeks compared with placebo, with a number needed to treat (NNT) of 3. During this period, it has similar efficacy to PE, oral prednisolone, and intravenous methylprednisolone. In one large trial, the benefit of IVIG persisted for 24 and possibly 48 weeks. Further research is needed to compare the long-term benefits as well as side effects of IVIG with other treatments (123).

In a recent PK study in 25 CIDP patients with active but stable disease serum IgG levels before and shortly after serial IVIG infusions were remarkably constant over time. The change in IgG levels was associated with IVIG dosage, but not with treatment frequency, and both inter- and intra-patient variability was low. This indicates that these patients have reached a steady state with a constant distribution rate and turnover of IgG without accumulation over time. Constant serum IgG levels seem to be required to stabilize CIDP patients (139). A study in two CIDP patients showed that weekly dosing with IVIG resulted in higher serum IgG trough levels, which correlated with improved clinical response (140). In a retrospective cohort study, 15 CIDP patients underwent successful gradual dose reductions. Most patient started on an initial dose of 2 g/kg/course and could reduce that dose by mean 63% at an average dose interval of 7 weeks (range of dose reduction: 42.4–88%; range of treatment frequency: 2–17 weeks). There was high variability between patients in observed IgG levels (141): the lowest effective dose of IVIG per course ranged between 18 and 108 g; it did not correlate to weight, frequency of administration, disease duration, or pre-therapeutic degree of disability. These results suggest considerably lower, standardized, initiating, and maintenance doses might be effective and highlight the need for prospective dose comparative trials (142).

Post-infusion rise in IgG levels (ΔIgG) were correlated in individual patients (*P* = 0.005), but inter-patient variability was high (142). No correlations were ascertained between IgG level variation and weight, BMI, functional improvement, total dose of IVIG administered, or dose of IVIG administered per kilogram per week. Required frequency of IVIG infusions may, however, relate to patient-specific post-infusion rise of ΔIgG levels hence possibly explaining inter-patient differences in treatment frequency needs. IgG level monitoring may be helpful in establishing optimum treatment regimens in individual cases (142).

The highly variable individual IgG doses and treatment intervals were also observed and analyzed by Broyeles et al. (143). The authors suggested that physicians might be adjusting IgG dosing in CIDP according to each patient’s clinical condition and treatment response. However, whether these adjustments will optimize clinical outcome while limiting overall costs has still to be seen. Not surprising that a Canadian cost-utility study using a Markov model failed to perceive IVIG as cost-effective treatment for CIDP compared to corticosteroid treatment (144). A criticism of this study may be that it was too short to capture all long-term problems encountered with corticosteroids. On the other hand, IVIG may be a short-term cost minimizing therapy compared to PE and in long-term maintenance therapy SCIG has been proven to be feasible, safe, effective, and cost reducing (145, 146). However, it should be stated that in Europe no application for a centralized authorization of a SCIG product in CIDP has as yet been submitted, while several IVIG products are authorized either nationally or via the MR or DC-procedures in numerous European countries supporting a class effect of current IVIG brands for the treatment of CIDP.

Nobile-Orazio et al. (132) compared in a RCT efficacy and tolerability of 6 month IVIG vs. IV methylprednisolone (0.5 g/day on four consecutive days given monthly for 6 months). Treatment of CIDP with IVIG for 6 months was less frequently discontinued because of inefficacy, adverse events, or intolerance than was treatment with IV methylprednisolone. The longer-term effects of these treatments on the course of CIDP need to be addressed in future studies, notably as in some patients improvement after corticosteroids seems to be more long-lasting than after IVIG (124).

Recent RCTs with rituximab (147), intramuscular interferon β-1a (148), and methotrexate (149) failed to show a beneficial effect as add-on therapy. In a review by Cocito et al. (150), analyzing 110 patients with refractory CIDP, various immunomodulatory drugs yielded similarly disappointing results. In contrast, small open-label studies investigating mycophenolate mofetil (151) and alemtuzumab (152) showed promising results on the possibility to stop or reduce maintenance IVIG therapy.

Better understanding of the pathogenesis is needed to identify new treatment strategies and to develop biomarkers that correlate with disease activity and could help guiding maintenance treatment in these patients.

### Multifocal motor neuropathy

Since the mid ‘80s MMN was identified as a treatable immune-mediated disease that responded to cyclophosphamide and IVIG (153, 154). A Cochrane Review (155) identified a total of 4 RCTs (including 34 patients) concerning the efficacy and safety of different IVIG brands in MMN. These showed that IVIG had a beneficial effect on strength and a non-significant trend toward improvement of disability. Two further open-label, non-controlled studies confirmed these results with 1 IVIG product in 20 MMN patients.

Currently, one IVIG product is licensed centrally in the entire EU; however, some other IVIG products are licensed nationally for MMN, supporting a class effect of current IVIG brands. Patients with stable clinical course on IVIG conditions can be safely switched to SCIG at the same monthly dose without risking deterioration but with an improvement of quality of life (156, 157).

**Table:** 

**Product specific indication and dosing:**
Multifocal Motor Neuropathy (MMN)
In general the starting dose is 2 g/kg for 2–5 days and the maintenance dose is 1 g/kg every 2–4 weeks or 2 g/kg every 4–8 weeks. IVIG may be switched to SCIG at the same monthly dose.
No current IVIG/SCIG coreSPC indication or dosing available.

#### Recent developments in MMN research

Multifocal motor neuropathy is a rare focal inflammatory neuropathy characterized by slowly progressive, asymmetric distal limb weakness without sensory loss. The hallmark of electrophysiological examination is a conduction block in the absence of abnormalities in sensory nerves. Differentiation from amyotrophic lateral sclerosis (ALS) and CIDP with asymmetric onset is important as these diseases differ in prognosis and treatment (153, 158). The underlying immuno-pathological mechanisms are unknown but IgM auto-antibodies against ganglioside GM1 and galactocerebroside GalC are thought to play a role (159).

First treatment option in MMN is IVIG. It improves muscle strength by 78% and to a lesser extent the disability (39%) in most patients (153). Corticosteroids, immunosuppressants, or PE are not effective therapies for MMN, actually these treatments can even worsen the paresis (158, 160, 161). Optimal dose and intervals in maintenance treatment have not been established. Evaluating serum IgG levels in MMN patients receiving a cumulative dose of 2.0 g/kg over 5 days, a wide variation was found in total IgG and ΔIgG levels between patients. Comparing IVIG responders with non-responders, the ΔIgG levels were higher in the IVIG responders at each time point (1, 5 days, and 3 weeks after treatment) with the largest difference on day 1 after IVIG (162). In several small studies (156, 157, 160), IVIG was switched to SCIG as maintenance therapy at the same monthly dose with beneficial results. An interesting dose-reduction protocol was described by Eftimov et al. (163) when switching from IVIG to SCIG in 10 stable-phase MMN patients: 5 received 100% of the IVIG maintenance dose, the other 5 were put on 50%. All patients in the lower dose group deteriorated.

In a small randomized trial, mycophenolate mofetil has been investigated as add-on therapy but did not show any additional effect over IVIG with placebo (164). Rituximab did not reduce the need for IVIG in another small open-label study in six MMN patients (165). Cyclophosphamide especially in combination with autologous stem cell transplantation has been used in clinical practice and is recommended in some guidelines for treatment of refractory patients (160).

### Myasthenia gravis (MG)

Myasthenia gravis is an autoimmune disease with auto-antibodies interfering with neuromuscular transmission. Auto-antibodies are directed to signaling proteins at the neuromuscular junction, in particular, the nicotinic acetylcholine receptor (AChR). At least three mechanisms have been proposed to explain how anti-AChR antibodies compromise neuromuscular transmission: (i) complement binding and activation at the neuromuscular junction; (ii) accelerated degradation of AChR molecules (antigenic modulation); and (iii) functional block of AChR-binding sites (166). As in other autoimmune neuropathies IVIG has been tried as therapy besides corticosteroid, immunosuppressants, and PE. The results for IVIG are less convincing than in MMN, CIDP, and GBS and currently, there is not sufficient solid data to include MG in the core SPC’s established indications.

Two small trials published in 1984 demonstrated that IVIG treatment was effective in MG patients at doses of 20 g given 6× over 2 weeks or 1–2 g/kg over 5 days (167). A study in 2005 compared 1 g/kg with 2 g/kg in MG, and found no significant difference between the two doses for the primary and secondary endpoints (168).

In the latest Cochrane systematic review, the authors analyzed all available RCTs (*n* = 7) differing in inclusion criteria and comparator treatment (169). The conclusion of this review is that there is no evidence from RCTs or from other trials to determine whether IVIG improves function or reduces the need for steroids.

### Fetal neonatal alloimmune thrombocytopenia

Fetomaternal or neonatal alloimmune thrombocytopenia is the most common cause of severe thrombocytopenia in an otherwise healthy fetus or neonate. Affected babies are at risk of bleeding, and ~10–20% (170, 171) may develop intra- and/or extra-uterine intracranial hemorrhage. In 1988, Bussel and colleagues (172) described the successful use of a weekly dose of 1 g/kg maternal weight starting when thrombocytopenia developed in the fetus until delivery, in seven pregnancies at risk. Meanwhile, several observational studies have reported on further successful cases in such affected women (173–176). Based on the results of the available data, there is no doubt that IVIG (starting between 12 and 20 weeks of gestation) is currently the standard therapy (171, 173, 174, 177). This indication is primarily based on the successful prevention of intracranial hemorrhage rather than on increasing the fetal platelet count. The question whether corticosteroids may have an additional positive effect in women treated with IVIG remains open (178).

From a regulatory perspective FNAIT would warrant further discussion.

#### Recent developments

Currently, different recombinant monoclonal anti-HPA-1a antibodies, which would be applied in the management of FNAIT, are under development in murine and translational models (179–182). In addition, efforts are now being made to establish a screening program that would help in identifying pregnancies at risk, and justify the prevention of immunization by vaccination or neutralization of the antibodies (180, 183).

### Fetal hemolytic disease

Immune antibodies to red blood cell (RBC) antigens can cause significant fetal anemia that may occur early in gestation, when fetal transfusion is difficult to perform. Based on the success observed in the treatment of FNAIT, IVIG has been used in cases with severe hemolysis that cannot be compensated by transfusion due to technical difficulties and/or highly aggressive antibodies. Although, the results are somewhat conflicting, some benefit was observed in most cases (184) using a weekly dose of 1 g/kg maternal weight, commencing from the first trimester. In a recent Cochrane study, the author’s conclusion was as follows: no information is available from randomized trials to indicate whether the antenatal use of IVIG is effective in the management of fetal RBC alloimmunization, although several case studies suggest that a beneficial role in delaying the onset of fetal anemia requiring invasive intrauterine transfusion (185).

### Dermatological diseases

Recommendations from the most recent European dermatology guideline for IVIG use list levels of evidence and grade of recommendations. These recommendations are briefly reiterated here. IVIGs were deemed to be efficacious in severe forms of dermatomyositis, polymyositis, and inclusion body myositis (184, 185) but are usually recommended as second-line therapy. The most convincing results have been reported in juvenile and adult myositis patients with acute, potentially life-threatening complications such as dysphagia, severe weakness, ulcerative skin leasions, and calcinosis cutis (188, 189). The authors further recommended IVIG as a second-line treatment in autoimmune blistering diseases, which are relapsing or refractory to standard therapy (190). In addition, for vasculitic syndromes with a particularly fulminant progressive disease form with multiple complications and severe side effects, IVIG therapy may be considered as a first-line treatment option. Less clear evidence exists for the use of IVIG in systemic lupus erythematosus. In numerous studies, the early administration of IVIG in toxic epidermal necrolysis (TEN) was suggested to be potentially life-saving (evidence level IV, recommendation grade C). The early administration of high-dose immunoglobulin should be considered in confirmed cases of TEN in the absence of any therapeutic alternative (187, 191).

For the various disorders, the authors recommended a dose of 2 g/kg body weight (3 g/kg in TEN) applied over a period of 2–5 days. For chronic dermatological diseases treatment, intervals should be every 4 weeks and after 6 months gradually increased to 6-week intervals.

## Discussion and Conclusion

Over the last 40 years, the demand of therapeutic Ig has been steadily increasing worldwide. This has several reasons (i) the number of indications kept extending from PID and SID into hematology, neurology, rheumatology, intensive care, and dermatology; by now international guidelines list 10–12 high-priority indications, 15–18 medium priority, and over 20 low-priority indications. (ii) IVIG is conceived as a safe, well-tolerated, and well-accepted medicine among patients and doctors explaining why it has been tried in so many different conditions and helped surprisingly quite often. (iii) The introduction of a variety of new preparations (5 and 10% IVIG and 16 and 20% SCIG, hyaluronidase facilitated SCIG and hyperimmune IVIG) and different routes of application (IV, SC with pump or rapid push, home treatments) has made the practical therapy more flexible and easier (8).

It is the purpose of this survey to review critically and to update indications and dosing strategies of IVIG and SCIG therapies in view of increasing demand, pharmacoeconomic aspects, and emerging alternatives for the immunomodulatory indications. Moreover, we wanted to recall the regulatory recommendations laid down in the core SPC of the European Medicine Agency and complement this information with current research and development data in the field of high-priority IVIG/SCIG replacement or immunomodulatory therapies.

Dosing of IVIG/SCIG in PAD replacement therapy no longer relies on fixed monthly doses but rather on a treat-to-target strategy the goal being a maximal reduction and control of bacterial infectious episodes and avoidance of side effects. Thus, monthly dosage, route of administration (IV or SC), infusion intervals and serum Ig levels are secondary to this goal but have to be optimized for the individual patient in order to reach the best possible result (24). The flexibility to comply with patients’ needs has been greatly improved. Not only can the same total weekly SCIG dose be administered at different intervals, from daily to biweekly (to monthly for the facilitated SCIG), with minimal impact on serum IgG levels (21, 22), SCIG and IVIG may also be applied in an alternating mode to increase convenience and quality of life (23). Several SCIG loading regimens rapidly achieve adequate serum IgG levels in treatment-naïve patients (22). There is reasonable evidence to calculate the loading dose on ideal body-weight (2, 3) and to increase monthly dosages by 0.15 g/kg until break-through infections are sufficiently controlled (17). Infusion-related reactions can be avoided or minimized in most cases by switching from IVIG to SCIG. While the FDA recommends the use of a DAC when switching from IVIG to SCIG (6, 7) the European experience reports satisfactory results by just maintaining the monthly IVIG dose and dividing it in weekly, biweekly, or even smaller doses (192). It is now generally accepted that Ig replacement therapy does more than just replace the antibody repertoire in PAD patients (30). It also serves as a biological response modifier with anti-inflammatory properties (31, 32) that contribute to the clinical benefit of IVIG/SCIG therapy in immunodeficient patients. The bottom line of the current knowledge on Ig replacement therapy is optimizing the results by individualizing the treatment regimens; recently, this principle has been beautifully illustrated by case studies from Bonagura (20).

Regarding the major immunomodulatory indications for IVIG, it is striking how little evidence and structure has so far been brought into the dosing issue. Most studies still start with the 2 g/kg protocol (preferably given on 1 single day, instead of being spread over 5 days), as originally described in the ITP (71, 72) and KD (87, 88) trials. Although a down-scaling regime in ITP showed that 1 g/kg was as efficient as 2 g/kg (72), it was the latter dose that set the stage for the regulatory adoption of the dosage regimen in ITP and other “immunomodulatory settings” for years to come. There are only a few case reports dictated by economic constraints suggesting that lower doses (0.1–0.4 g/kg) may also exhibit effective immunomodulatory activity (77, 78) but no systematic dose exploration has been performed. Especially no up-scaling dosage regimen has been pursued as in Ig replacement therapy for PAD patients (17). Down-scaling attempts (141, 142) and a trend to switch from IVIG to SCIG have been made in chronic diseases such CIDP (145, 146) and MMN (156, 157, 160, 163). In the acute phases of GBS or in myasthenic crisis comparative studies between PE and IVIG showed equal effectiveness but advantages for IVIG with respect to feasibility and costs. Despite much effort that has been put into the analysis of mechanisms governing responsiveness and non-responsiveness to high-dose IVIG in ITP, KD, and the neuroimmunological indications no clear-cut picture has emerged so far (186). Some biomarkers, however, may be helpful to identify responders from non-responders and thereby try early on alternative treatment options (94–96, 111). Driven by the need to be cost-effective the pursuit of alternative treatment options is another strong trend in many studies on immunomodulatory IVIG indications. Thus, the renaissance of corticosteroids in the long-term treatment of KD (97, 103–105) and CIDP (132, 135) are examples. A recent overview of immunological treatment options in neuroimmunological emergencies by von Geldern et al. (193) nicely illustrates that IVIG is only one among several treatment choices, although a very important and effective one.

Clearly, more research is needed to clarify the mode of action of IVIG in immunomodulation and to optimize dosing and treatment intervals.

## Conflict of Interest Statement

Jacqueline Kerr, none; Isabella Quinti, is a member of the Baxter and CSL-Behring advisory boards; Helen Chapel is a consultant for Biotest and LFB and has worked as a consultant for Baxter Healthcare, Grifols, and CSL-Behring; Martha Eibl, none; Hans-Hartmut Peter is member of the Pfizer scientific advisory board of the pneumococcal conjugate vaccine PCV13 program in Germany. Occasional lecturer for CSL-Behring and Novartis Ag. Abdulgabar Salama, none; Ivo N. van Schaik, received departmental honoraria for serving on scientific advisory boards for CSL-Behring and Baxter and a steering committee for CSL-Behring; Peter J. Späth, none; W. A. Carrock Sewell, none; Taco W. Kuijpers, none.

## Footnotes

^1^http://www.ema.europa.eu/ema/index.jsp?curl=pages/medicines/landing/epar_search.jsp&mid=WC0b01ac058001d124

^2^http://mri.medagencies.org/Human/

^3^http://www.ema.europa.eu/ema/index.jsp?curl=pages/medicines/human/medicines/002491/human_med_001647.jsp&mid=WC0b01ac058001d124

^4^http://www.ema.europa.eu/ema/index.jsp?curl=pages/medicines/human/medicines/002127/human_med_001440.jsp&mid=WC0b01ac058001d124
